# Supplementary material for: Intermediate soil acidification induces highest nitrous oxide emissions
Source: Nat Commun. 2024 Mar 27;15:2695. doi: 10.1038/s41467-024-46931-3 (PMC10973416; doi:10.1038/s41467-024-46931-3)
Supplement: Supplementary file 1 — Supplementary Information [file 41467_2024_46931_MOESM1_ESM.pdf]

Supplementary information for

Intermediate soil acidification induces highest nitrous oxide emissions

Yunpeng Qiu[1], Yi Zhang[1], Kangcheng Zhang[1], Xinyu Xu[1], Yunfeng Zhao[1], Tongshuo Bai[1], Yexin Zhao[1], Hao Wang[1], Xiongjie Sheng[1,2], Sean Bloszies[3], Christopher J. Gillespie[3], Tangqing He[1], Yang Wang[4], Huaihai Chen[5], Lijin Guo[6], He Song[7], Chenglong Ye[1], Yi Wang[8], Alex Woodley[9], Jingheng Guo[10], Lei Cheng[11], Yongfei Bai[4], Yongguan Zhu[12,13,14], Sara Hallin[15], Mary K. Firestone[16,17], Shuijin Hu[3,1*]

[1]College of Resources and Environmental Sciences, Nanjing Agricultural University, Nanjing 210095, China.

[2]School of Ecology and Environmental Sciences, Yunnan University, Kunming 650091, China.

[3]Department of Entomology & Plant Pathology, North Carolina State University, Raleigh, NC 27695, USA.

[4]State Key Laboratory of Vegetation and Environmental Change, Institute of Botany, Chinese Academy of Sciences, Beijing 100093, China.

[5]State Key Laboratory of Biocontrol, School of Ecology, Shenzhen Campus of Sun Yat-sen University, Shenzhen, Guangdong 518107, China.

[6]International Magnesium Institute, College of Resources and Environment, Fujian Agriculture and Forestry University, Fuzhou 350002, China.

[7]College of Agronomy, Anhui Agricultural University, Hefei 230036, China.

[8]State key Laboratory of Loess and Quaternary Geology, Institute of Earth Environment, Chinese Academy of Sciences, Xi'an 710061, China.

[9]Department of Crop and Soil Sciences, North Carolina State University, Raleigh, NC 27695, USA.

[10]Beijing Key Laboratory of Farmland Soil Pollution Prevention and Remediation, College of Resources and Environmental Sciences, China Agricultural University, Beijing 100193, China.

[11]MOE Key Laboratory of Biosystems Homeostasis & Protection, College of Life Sciences, Zhejiang University, Hangzhou 310058, China.

[12]Key Laboratory of Urban Environment and Health, Institute of Urban Environment, Chinese Academy of Sciences, Xiamen 361021, China.

[13]State Key Laboratory of Environmental Chemistry and Ecotoxicology, Research Center for Eco-Environmental Sciences, Chinese Academy of Sciences, Beijing 100049, China.

[14]Zhejiang Key Laboratory of Urban Environmental Processes and Pollution Control, CAS Haixi Industrial Technology Innovation Center in Beilun, Ningbo 315830, China.

[15]Department of Forest Mycology and Plant Pathology, Swedish University of Agricultural Sciences, Uppsala 75007, Sweden.

[16]Department of Environmental Science, Policy, and Management, University of California, Berkeley, Berkeley, CA 94720, USA.

[17]Earth and Environmental Sciences, Lawrence Berkeley National Laboratory, Berkeley, CA 94720, USA.

[*]Corresponding author: shuijin_hu@ncsu.edu

The PDF file includes:

Supplementary Notes

Supplementary Figs. 1 to 16

Supplementary Tables 1 to 7

Supplementary Notes

1. Different patterns in the relationship between soil pH vs. coarse EFs and soil pH vs. averaged EFs

The different patterns of the relationship between soil pH vs. coarse and averaged EFs primarily stem from the fact that these two methods give different statistical weight for each soil pH increment. In the pH-coarse EF approach, a pH increment with more data points (i.e., field measurements) (e.g., pH6.0, 153 data points) is given much higher weight than the pH increment with fewer data points (e.g., pH4.5, 14 data points). Consequently, the statistical analysis is highly skewed towards the pH increments with large numbers of field experiments and measurements, leading to a linear regression explaining a very low proportion of the data variation. However, this does not provide a fair assessment of pH effect on N_2O EFs. The averaged method solves this issue by averaging data points at each pH increment to obtain one EF and then giving all the pH increments equal weights. Our work is the first to address this issue and, for the first time, identified the unique relationship between pH and N_2O EFs that has previously been overlooked. This issue might have been overlooked in a couple of previous meta-analyses that only showed a weak linear relationship[1] or no relationship[2]. One major reason that leads to the overlook may be due to the scale-issue in the diagram. Because some N_2O EFs were much higher than others, the Y axis in the coarse-pH diagram needs to be set much higher than the average, in order to cover all the data points, which visually gives a false impression of linear relationship. If we reduce the Y-axis scale in the pH-coarse EF diagram to the same range for the pH-averaged EF diagram, we can visually see that both the pH-coarse curve and the pH-averaged curve were similar and in hump-shape (see Supplementary Fig. 5 below). However, explanation power of the variation was greatly different

because the coarse method incorrectly gives well more weight to the pH point with more measurements, as we described in the Main text.

2. Why were three grasslands as model systems selected for our field pH manipulation experiments

We selected three grasslands for our field pH manipulations to assess the relationship between soil pH and N_2O emissions for three major reasons:

First, our primary purpose of the field manipulative experiments was to examine how acidification (soil pH) alone affects N_2O EFs and N_2O emissions. Input of N fertilizers (often in the form NH_4^+) directly affects N_2O EFs and emissions by increasing N substrates, but also indirectly affects them through soil acidification, leading to the confounding effect of available N and soil pH[1,3]. Croplands in China have received high amounts of N fertilization[4,5] and soil pH in the major Chinese crop-production areas, on average, has declined significantly by ca. 0.5 unit from the 1980s to the 2000s[4]. Also, plant harvest removes other nutrients from agricultural soils and agricultural practices (e.g., tillage and fungicides) disturb soils, which could have other effects on N_2O EFs and emissions. Isolation of acidification from other human-induced effects on N-cycling microbes and N_2O EFs is one major reason why we did not use agricultural soils.

Second, applications of N fertilizers modify the community composition of N-cycling microbes (both nitrifiers and denitrifiers)[6] and the selective pressure of N fertilizer inputs on the functional characteristics of soil microbial communities may mask the effects of soil pH on N-cycling microbes and N_2O emissions[3]. Since none of the experimental sites had received any significant reactive N input (N deposition or N fertilizers), the selection pressure of human-derived N on soil N-cycling microorganisms was negligible.

Third, we want to have field experiments on acidic, neutral and alkaline soils that also have decent amounts of available soil N. Available soil N (particularly NO_3^-) in other unfertilized soils, like forest soils, is very low and likely constrains N-cycling microbes[7].

Moreover, grasslands cover about 40% of the global land surface and are widely used as pasture, supporting the livelihoods of more than 1.3 billion people through livestock production[8,9]. Global grasslands potentially contribute ca. 20% of total N_2O flux to the atmosphere[10,11]. Globally, there is a considerable proportion of grasslands under moderate to intensive management with fertilization and it is expected that more grasslands will be under fertilization in the future[11,12].

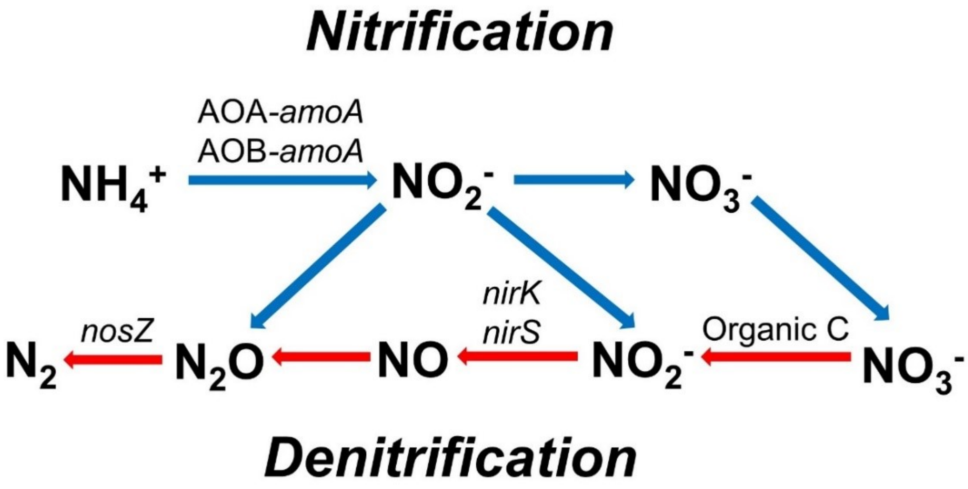

Supplementary Fig. 1. A simplified diagram summarizing nitrification and denitrification processes and the dominating genes that encode the related enzymes. AOA-*amoA* and AOB-*amoA* represent archaeal and bacterial *amoA* genes, respectively. *nirK*, *nirS* and *nosZ* represent genes encoding the Cu-containing nitrite reductases, cytochrome cd_1 nitrite reductase, and the nitrous oxide reductases, respectively.

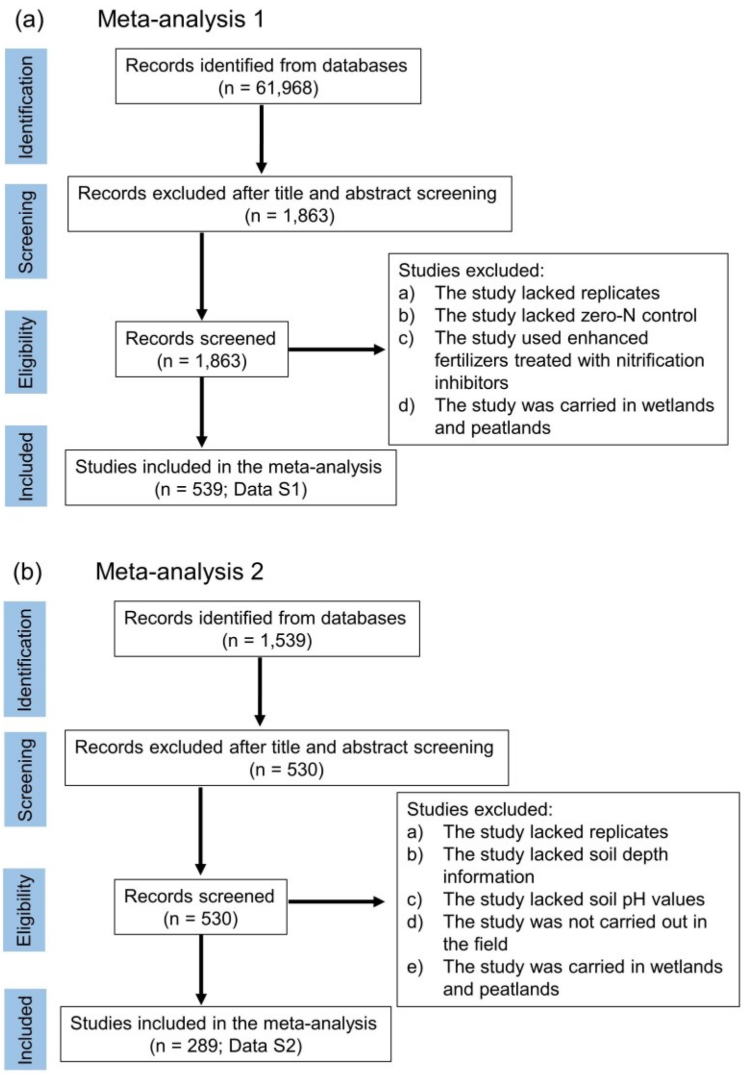

Supplementary Fig. 2. PRISMA Flow Diagram for the dataset of meta-analysis 1 (a) and 2 (b).

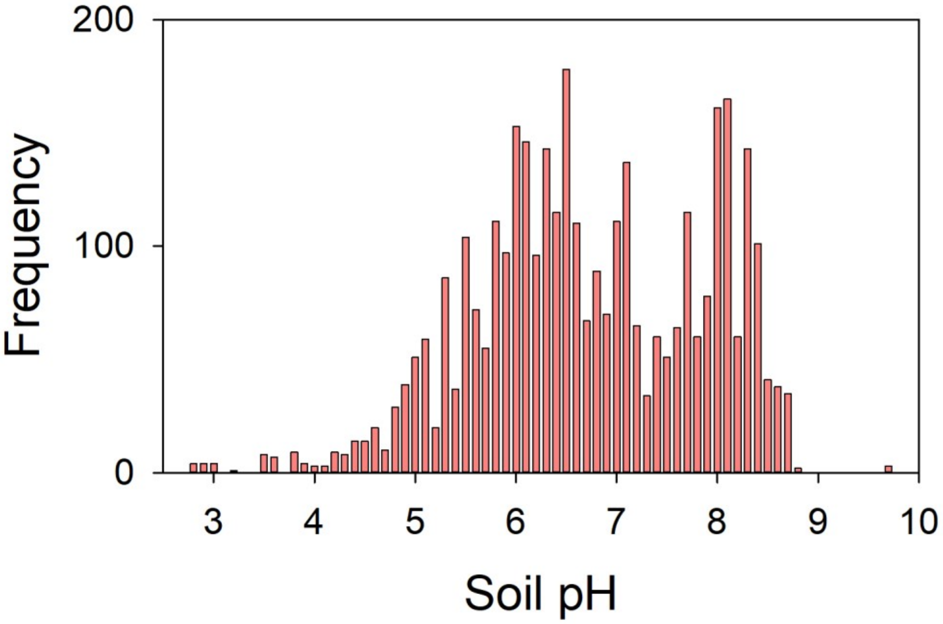

Supplementary Fig. 3. Data distributions of soil pH (determined in H_2O solution) in the meta-analysis of fertilizer N and pH effects on N_2O EFs (Meta-analysis 1). Source data are provided as a Source Data file.

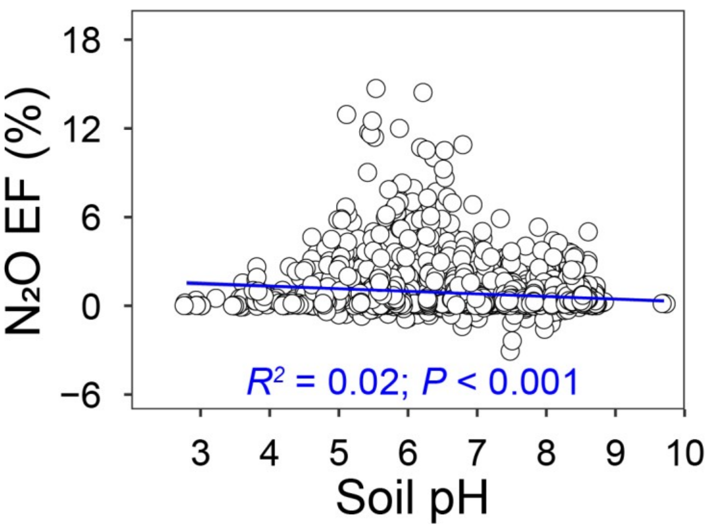

Supplementary Fig. 4. Linear relationship between soil pH and coarse N_2O EFs (Meta-analysis 1). Linear regression model with two-sided test was used for the statistical analysis (n = 3,562). The error band (shaded area) represents the 95% confidence intervals around the linear regression line. Statistics (adjusted R^2 and P values) for linear regression are indicated. Linear and quadratic model selection is shown in Supplementary Table 3. Source data are provided as a Source Data file.

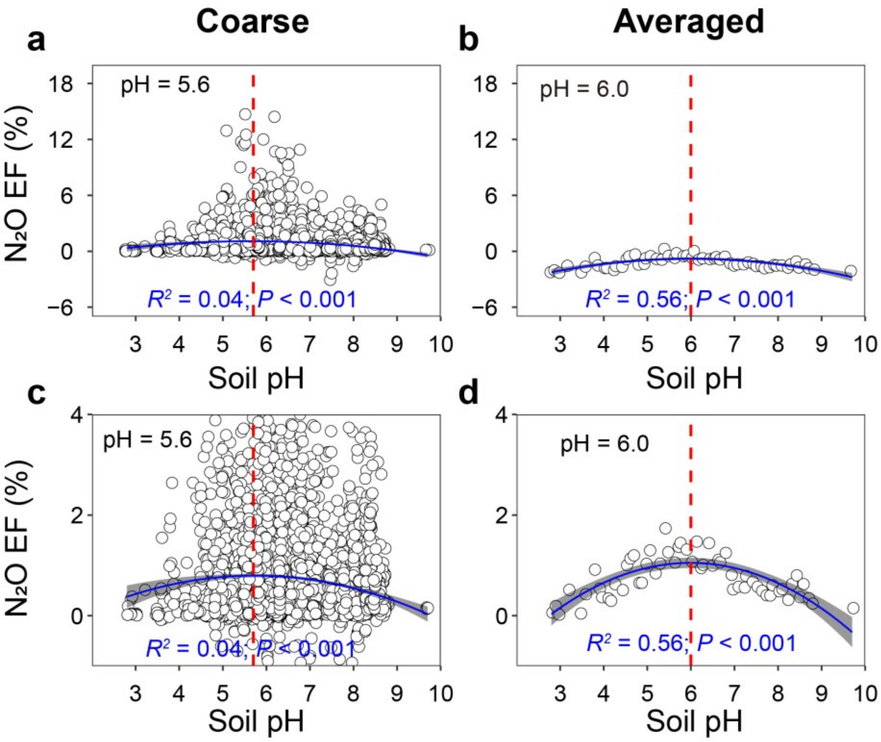

Supplementary Fig. 5. Relationship between soil pH and coarse EFs or averaged EFs with the same y axis range [a, b (from -7 to 20) or c, d (from -1 to 4)]. Linear regression model with two-sided test was used for the statistical analysis (n = 3,562 in **a, c**; n = 58 in **b, d**). The error bands (shaded areas) represent the 95% confidence intervals around the best-fit regression line. Statistics (adjusted R^2 and P values) for polynomial regression are indicated. The exact P values: $P < 0.001$ in **a-d.** Source data are provided as a Source Data file.

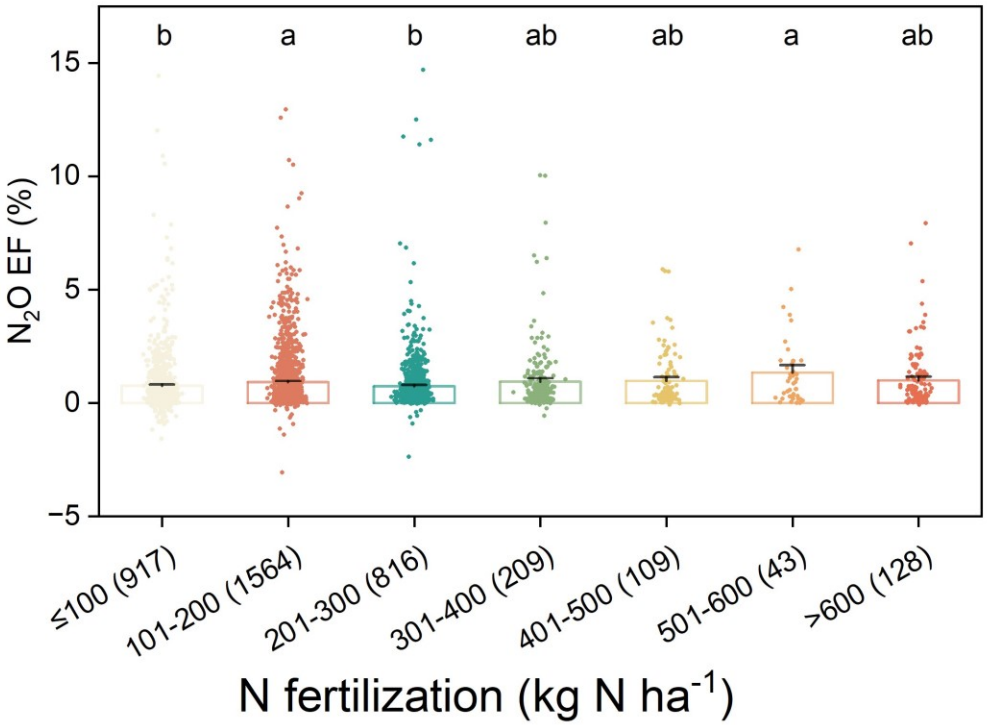

Supplementary Fig. 6. Non-parametric Wilcoxon test of soil N_2O EFs among different N fertilization groups in the meta-analysis of fertilizer N and pH effects on N_2O EFs. Bars represent mean ± s.e.m. Numbers in brackets on the x-axis indicate the number of independent measurements. Different letters indicate significant difference at $P < 0.05$. The exact P value: $P < 0.001$. Source data are provided as a Source Data file.

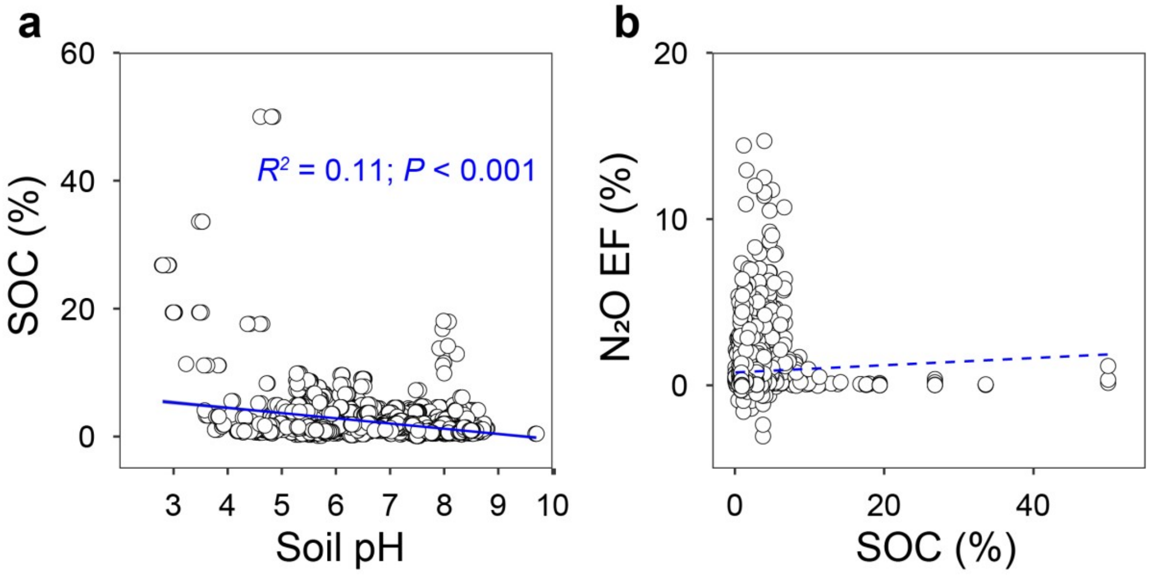

Supplementary Fig. 7. Relationship between soil organic C (SOC) and soil pH (a), and N_2O EFs and SOC (b) in the meta-analysis of fertilizer N effects on N_2O EFs. Linear regression model with two-sided test was used for the statistical analysis (n = 3,465 in **a** and n = 3,596 in **b**). The error band (shaded area) in **a** represents the 95% confidence intervals around the linear regression line, and the blue dashed line in **b** indicates an insignificant relationship. Statistics (adjusted R^2 and P values) for linear regression are indicated. Source data are provided as a Source Data file.

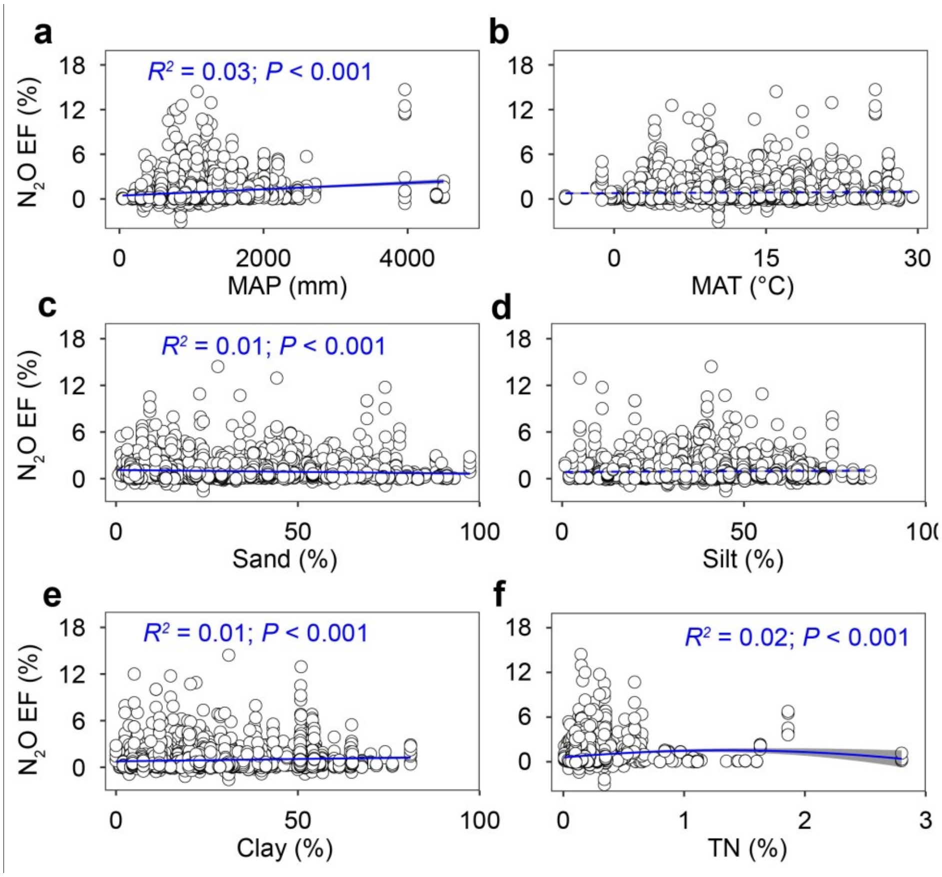

Supplementary Fig. 8. Relationship between mean annual precipitation (MAP, a), temperature (MAT, b), soil sand (c), silt (d) and clay content (e), and total soil nitrogen content (f), and N_2O EFs in the meta-analysis of fertilizer N effects on N_2O EFs. Linear regression model with two-sided test was used for the statistical analysis (n = 3,786 in **a**, n = 2,377 in **c,** n = 2,829 in **e**, and n = 2,825 in **f**). The error bands (shaded areas) in **a**, **c**, **e**, **f** represent the 95% confidence intervals around the linear regression line, and the blue dashed line in **b**, **d** indicates an insignificant relationship. Statistics (adjusted R^2 and P values) for linear regression are indicated. Source data are provided as a Source Data file.

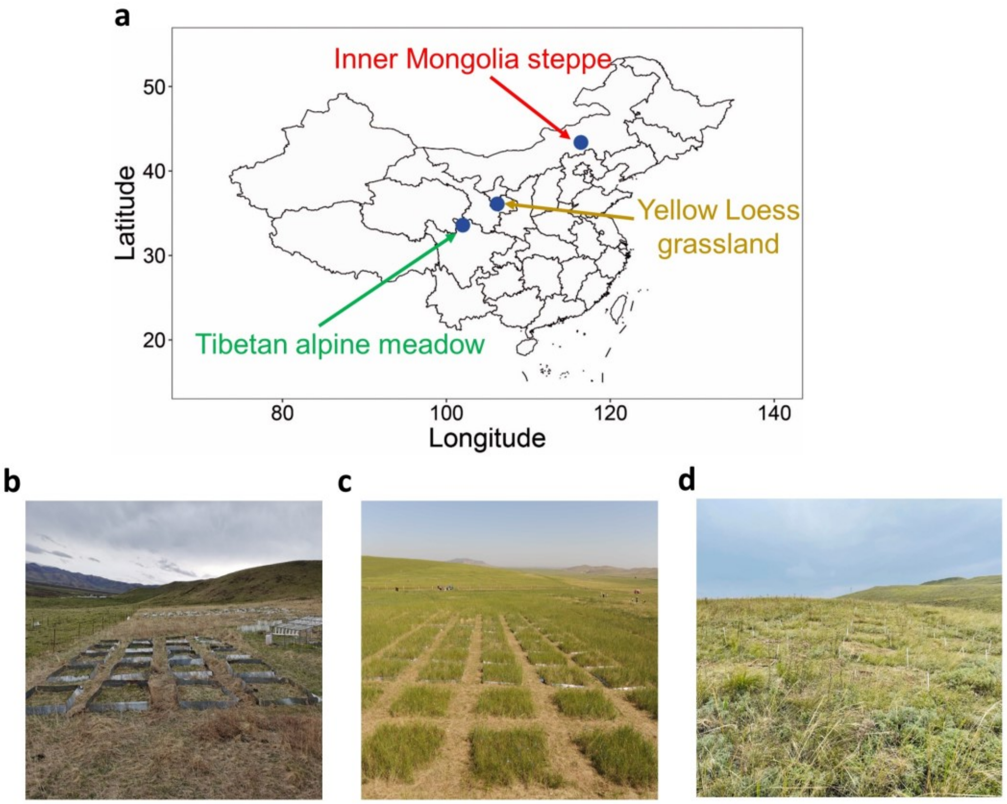

Supplementary Fig. 9. Geographical distribution of sampling sites (a), and overviews of field acid addition experiments in the Gannan Alpine Meadow (b), the Inner Mongolia steppe (c) and the Guyuan semi-arid grassland on the Loess Plateau (d). Soil sampling locations in the Gannan Alpine Meadow, the Inner Mongolia steppe and the Guyuan semi-arid grassland on the Loess Plateau are created using maptools [https://CRAN.R-project.org/package=maptools] package under the R environment software. Photo credit: Yunpeng Qiu (**b**, **d**) and Yongfei Bai (**c**)**.**

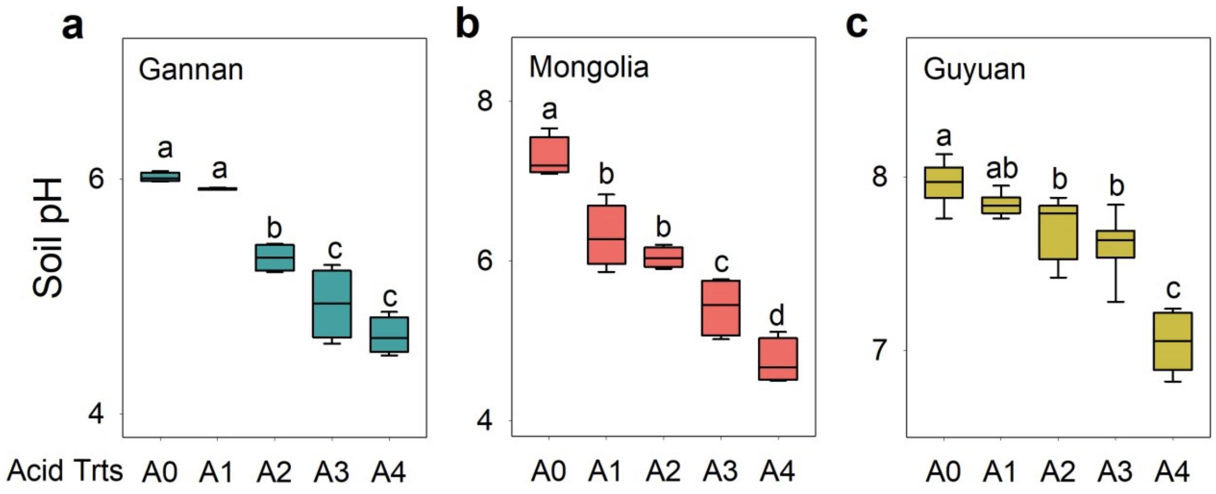

Supplementary Fig. 10. Effects of acid additions on soil pH in the Gannan alpine meadow (a), the Inner Mongolia steppe (b), and the Guyuan semi-arid grassland (c). Acid treatments correspond to A0, A1, A2, A3 and A4. One-way ANOVA with two-sided and post-doc test was conducted to determine significant differences. Different letters indicate significant differences among the levels of acid addition at $P < 0.05$. The box plots show the upper and lower quartiles (box limits), median (centre line), and the whiskers extend to a maximum of 1.5 times the interquartile range (IQR). For **a, b,** n = 4; **c,** n = 6. The exact P values: $P < 0.001$ in **a-c**. Source data are provided as a Source Data file.

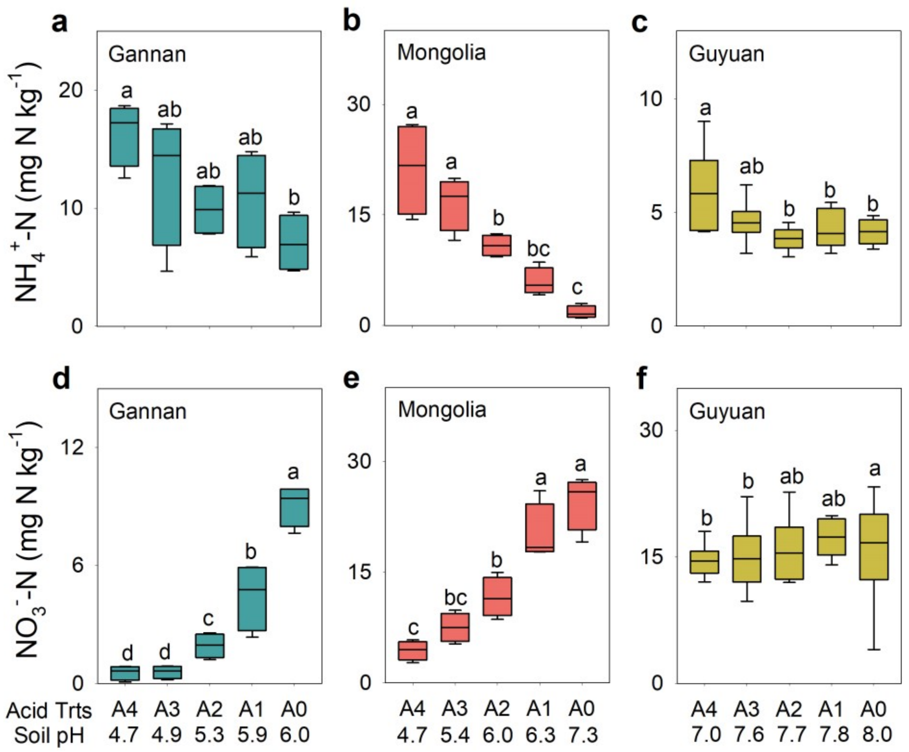

Supplementary Fig. 11. Effects of acid additions on soil extractable NH_4^+-N (a-c) and NO_3^--N (d-f) in the Gannan alpine meadow (a, d), the Inner Mongolia steppe (b, e), and the Guyuan semi-arid grassland (c, f). One-way ANOVA with two-sided and post-doc test was conducted to determine significant differences. Different letters indicate significant differences among the levels of acid addition at $P < 0.05$. The box plots show the upper and lower quartiles (box limits), median (centre line), and the whiskers extend to a maximum of 1.5 times the interquartile range (IQR). For **a, b, d, e,** n = 4; **c, f,** n = 6. The exact *P* values: $P = 0.028$ in **a,** $P < 0.001$ in **b, d, e**, $P = 0.004$ in **c,** and $P = 0.030$ in **f**. Source data are provided as a Source Data file.

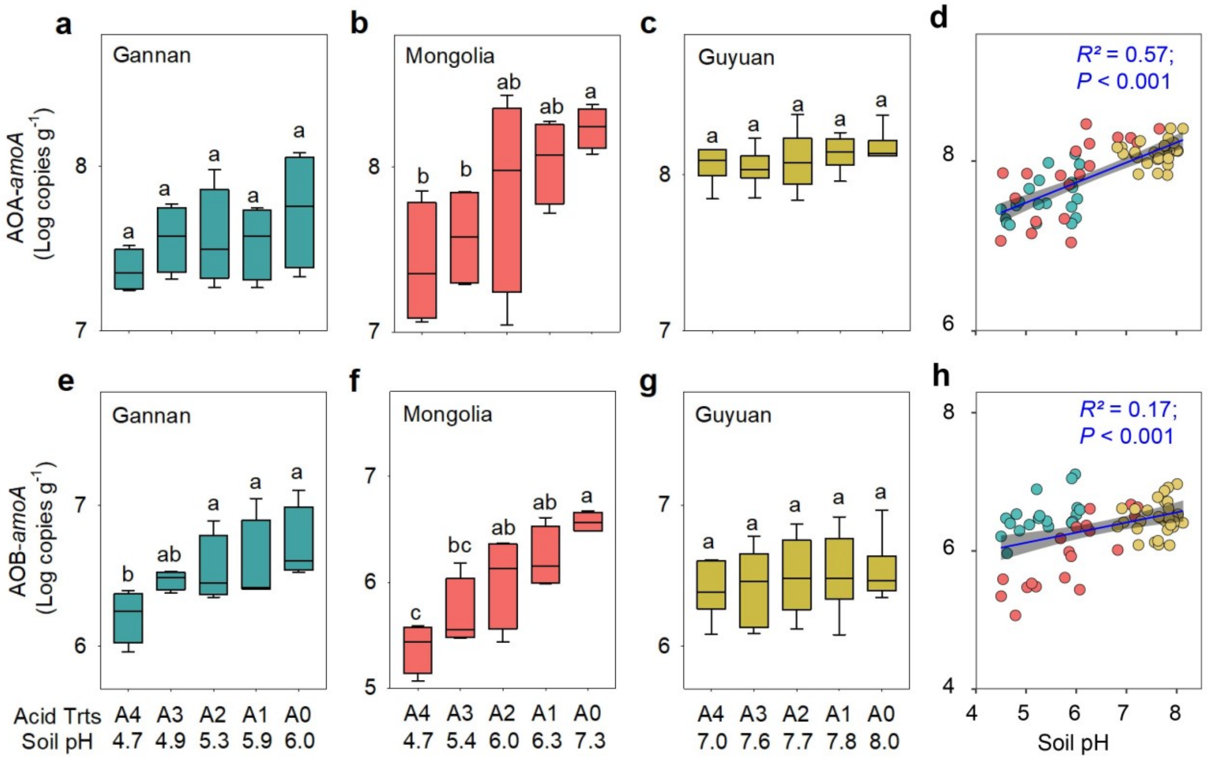

Supplementary Fig. 12. Effects of acid additions on soil AOA (a-d) and AOB (e-h) in the Gannan alpine meadow (a, e), the Inner Mongolia steppe (b, f) and the Guyuan semi-arid grassland (c, g), respectively. One-way ANOVA with two-sided and post-doc test was conducted to determine significant differences. Different letters indicate significant differences among the levels of acid addition at $P < 0.05$ in **a-c** and **e-g**. The box plots show the upper and lower quartiles (box limits), median (centre line), and the whiskers extend to a maximum of 1.5 times the interquartile range (IQR). For **a, b, e, f,** n = 4; **c, g,** n = 6. The exact P values: $P = 0.420$ in **a,** $P = 0.031$ in **b,** $P = 0.416$ in **c,** $P = 0.014$ in **e,** $P < 0.001$ in **f** and $P = 0.746$ in **g**. In **d**, **h**, linear regression model with two-sided test was used for the statistical analysis (n = 70 in **d**, **h**). The error bands (shaded areas) in **d, h** represent the 95% confidence intervals around the linear regression line. Statistics (adjusted R^2 and P values) for linear regression are presented. Source data are provided as a Source Data file.

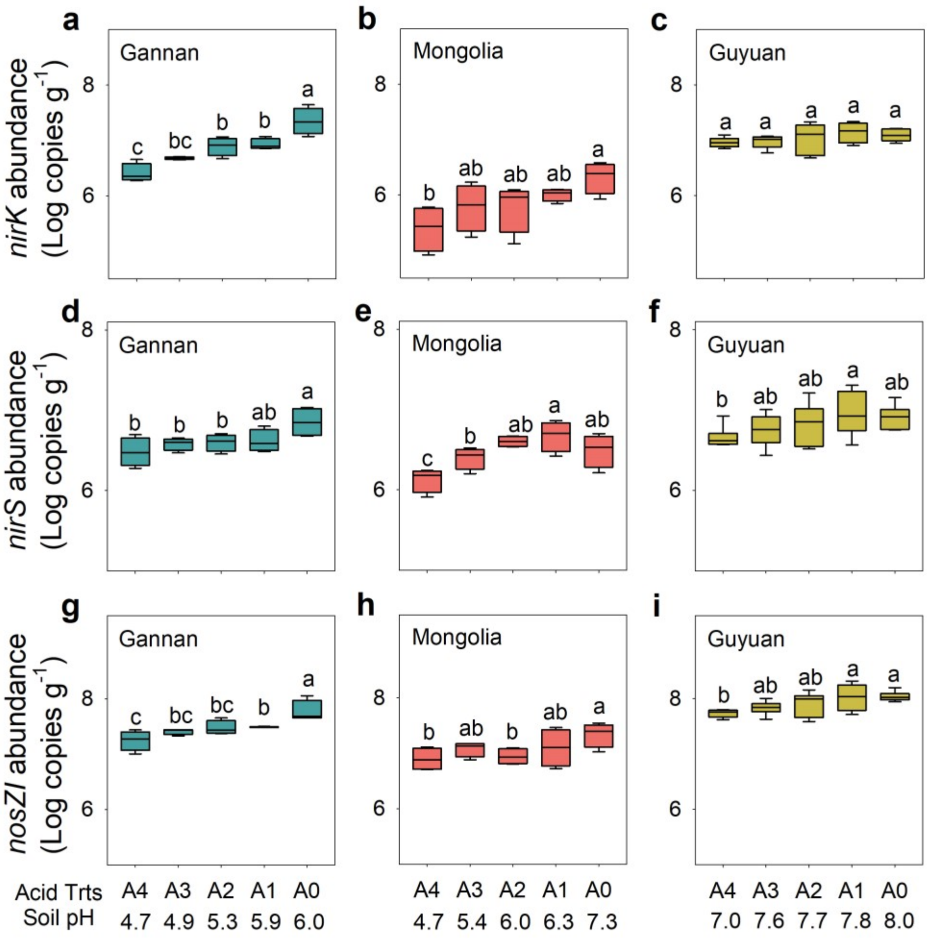

Supplementary Fig. 13. Effects of acid additions on soil *nirK*- (a-c), *nirS*- (d-f), and *nosZI*-type denitrifiers (g-i) in the Gannan alpine meadow (a, d, g), the Inner Mongolia steppe (b, e, h) and the Guyuan semi-arid grassland (c, f, i), respectively. One-way ANOVA with two-sided and post-doc test was conducted to determine significant differences. Different letters indicate significant differences among the levels of acid addition at $P < 0.05$. The box plots show the upper and lower quartiles (box limits), median (centre line), and the whiskers extend to a maximum of 1.5 times the interquartile range (IQR). For **a, b, d, e, g, h,** n = 4; **c, f, i,** n = 6. The exact P values: $P < 0.001$ in **a, e, g,** $P = 0.032$ in **b,** $P = 0.087$ in **c,** $P = 0.014$ in **d,** $P = 0.004$ in **f,** $P = 0.011$ in **h,** and $P = 0.002$ in **i**. Source data are provided as a Source Data file.

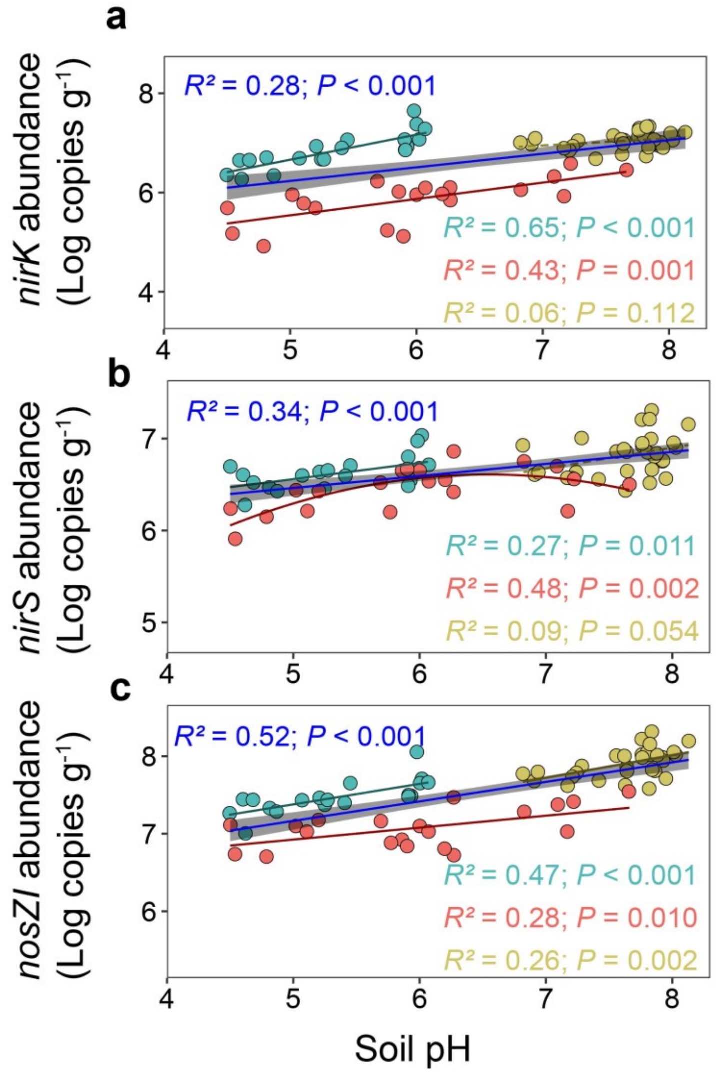

Supplementary Fig. 14. Relationships between soil pH and the abundance of *nirK*- (a), *nirS*- (b), and *nosZI*-type denitrifiers (c) in the Gannan alpine meadow, the Inner Mongolia steppe and the Guyuan semi-arid grassland, respectively. Linear regression model with two-sided test was used for the statistical analysis. Cyan, red, yellow, and blue lines represent correlations in Gannan (n = 20), Inner Mongolia (n = 20), Guyuan (n = 30), and three sites combined (n = 70), respectively. The error bands (shaded areas) represent the 95% confidence intervals around the linear regression line. Statistics (adjusted R^2 and P values) for linear and polynomial regression are presented. Source data are provided as a Source Data file.

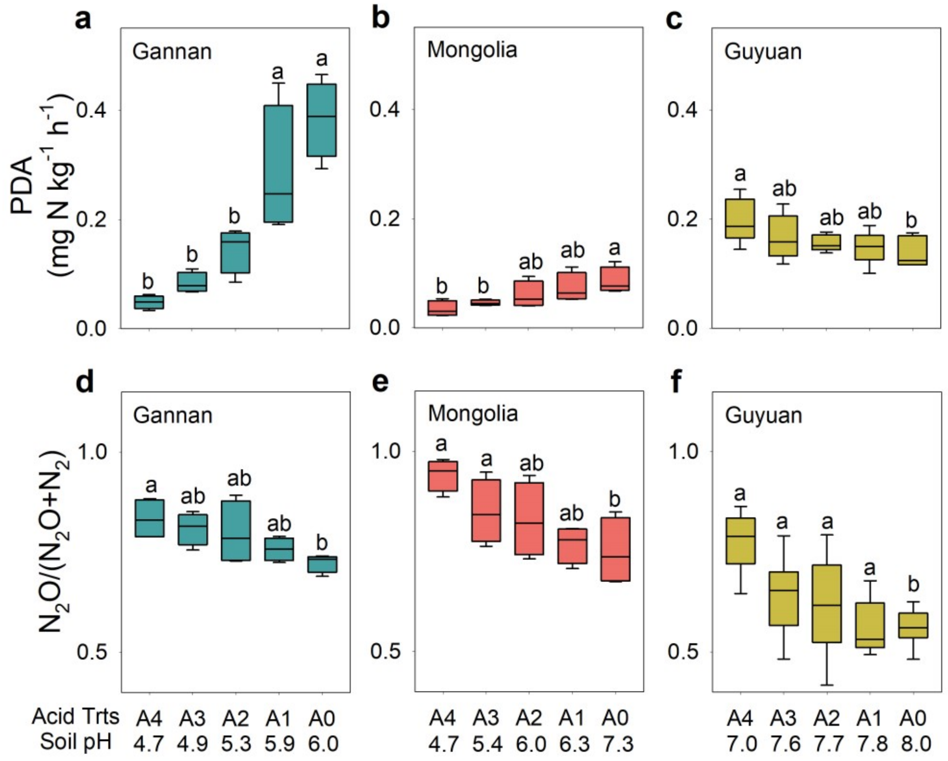

Supplementary Fig. 15. Effects of acid additions on soil potential denitrification activity (PDA) and $N_2O/(N_2O+N_2)$ product ratio of denitrification in the Gannan alpine meadow (a, d), the Inner Mongolia steppe (b, e), and the Guyuan semi-arid grassland (c, f). One-way ANOVA with two-sided and post-doc test was conducted to determine significant differences. Different letters indicate significant differences among the levels of acid addition at $P < 0.05$. The box plots show the upper and lower quartiles (box limits), median (centre line), and the whiskers extend to a maximum of 1.5 times the interquartile range (IQR). For **a, b, d, e,** n = 4; **c, f,** n = 6. The exact P values: $P < 0.001$ in **a, f,** $P = 0.024$ in **b,** $P = 0.038$ in **c,** $P = 0.049$ in **d,** and $P = 0.014$ in **e**. Source data are provided as a Source Data file.

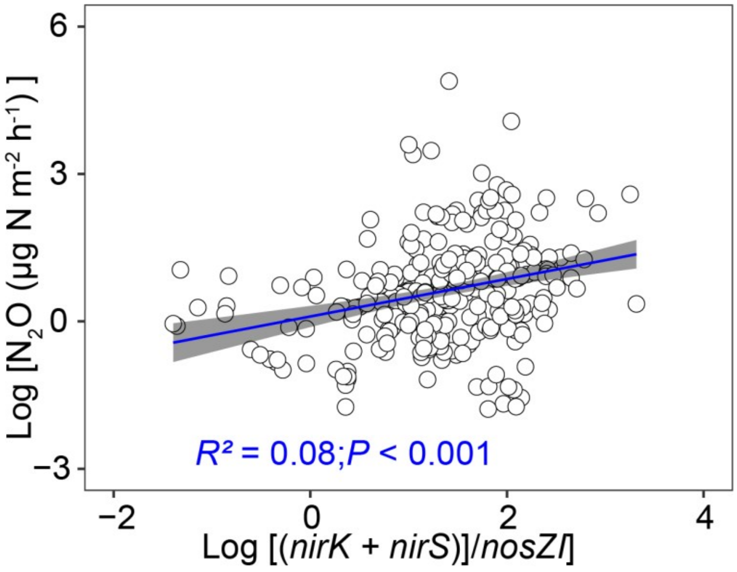

Supplementary Fig. 16. Relationship between N_2O emissions and the (*nirK*+*nirS*)/*nosZI* ratio in the meta-analysis of pH effects on denitrifying microbes. Linear regression model with two-sided test was used for the statistical analysis (n = 323). The error band (shaded area) represents the 95% confidence intervals around the linear regression line. Statistics (adjusted R^2 and P values) for linear regression are presented. Source data are provided as a Source Data file.

Supplementary Table 1. Vegetation types, climate conditions, and basic soil physical and chemical properties of the three experimental sites (also see Supplementary Fig. 8).

Location	Gannan	Inner Mongolia	Guyuan
Grassland type	Alpine meadow	Steppe meadow	Semi-arid grassland
Altitude (m)	3538	1250	1800
MAT (°C)	1.2	0.3	7.0
MAP (mm)	615	346	425
$pH_{(H_2O)}$	6.0	7.3	8.0
TC (%)	9.8	2.3	4.0
TN (%)	0.7	0.2	0.4
MBC (mg C kg^{-1})	995	802	824
MBN (mg N kg^{-1})	223	109	114

MAT, mean annual temperature; MAP, mean annual precipitation; TC, soil total C; TN, soil total N; MBC, microbial biomass C; MBN, microbial biomass N.

Supplementary Table 2. Soil acidity or alkalinity was divided into the following classes following the Soil Science Division Staff (2017).

Denomination	pH range
Ultra-acidic	<3.5
Extremely acid	3.5-4.4
Very strongly acid	4.5-5.0
Strongly acid	5.1-5.5
Moderately acid	5.6-6.0
Slightly acid	6.1-6.5
Neutral	6.6-7.3
Slightly alkaline	7.4-7.8
Moderately alkaline	7.9-8.4
Strongly alkaline	8.5-9.0

Supplementary Table 3. Summary for the linear and quadratic models predicting the relationship between N_2O EFs and soil pH, and N fertilization rate in the meta-analysis of synthetic N and pH effects on soil N_2O EFs (Meta-analysis 1). Linear regression model with two-sided test was used for the statistical analysis, and adjusted R-squared was used. Models are ranked by significance, AICc and parsimony. AICc measures the relative goodness of fit of a given model; the lower its value, the more likely the model is correct. ΔAICc denotes the difference between the AICc of each model and that of the best model.

	Model	Adjuested R^2	*P* value	AICc	Delta AICc	Selected Model
Soil pH vs.	Linear	0.022	<0.001	11972.9	43.92	
EFs	Quadratic	**0.035**	**<0.001**	**11928.9**	**0.00**	✔
N rate vs.	Linear	0.001	0.061	12780.7	0.88	**undetermined**
EFs	Quadratic	0.002	0.041	12779.8	0.00	
MAP vs.	Linear	**0.028**	**<0.001**	**12677.5**	**1.07**	✔
EFs	Quadratic	0.028	<0.001	12676.5	0.00	
Sand vs.	Linear	**0.006**	**<0.001**	**8072.8**	**0.00**	✔
EFs	Quadratic	0.006	<0.001	8074.4	1.57	
Clay vs.	Linear	**0.005**	**<0.001**	**9530.9**	**0.00**	✔
EFs	Quadratic	0.005	<0.001	9532.8	1.97	
TN vs.	Linear	0.011	<0.001	9169.6	14.6	
EFs	Quadratic	**0.017**	**<0.001**	**9155.0**	**0.00**	✔

Supplementary Table 4. Summary for the linear and quadratic models predicting the relationship between N_2O averaged EFs and soil pH in the meta-analysis of synthetic N and pH effects on soil N_2O EFs (Meta-analysis 1). Linear regression model with two-sided test was used for the statistical analysis, and adjusted R-squared was used. Models are ranked by significance, AICc and parsimony. AICc measures the relative goodness of fit of a given model; the lower its value, the more likely the model is correct. ΔAICc denotes the difference between the AICc of each model and that of the best model.

	Model	Adjuested R^2	*P* value	AICc	Delta AICc	Selected Model
Soil pH vs. EFs	Linear	0.000	0.883	66.4	46.98	
	Quadratic	**0.557**	**<0.001**	**19.4**	**0.00**	✔

Supplementary Table 5. Summary for the linear and quadratic models predicting the relationship between soil pH and N_2O emissions, (*nirK*+*nirS*)/*nosZI* ratio, PDA, and the $N_2O/(N_2O+N_2)$ product ratio of denitrification across the three field experiments. Linear regression model with two-sided test was used for the statistical analysis, and adjusted R-squared was used. Models are ranked by significance, AICc and parsimony. AICc measures the relative goodness of fit of a given model; the lower its value, the more likely the model is correct. ΔAICc denotes the difference between the AICc of each model and that of the best model.

	Model	Adjusted R^2	*P* value	AICc	Delta AICc	Selected Model
N_2O emissions	Linear	0.191	<0.001	143.2	25.5	
	Quadratic	**0.448**	**<0.001**	**117.7**	**0.00**	✔
(*nirK*+*nirS*)/*nosZI*	Linear	0.173	**<0.001**	-91.5	31.44	
	Quadratic	**0.482**	**<0.001**	**-122.9**	**0.00**	✔
PDA	Linear	0.050	0.035	-130.2	8.49	
	Quadratic	**0.173**	**<0.001**	-138.7	**0.00**	✔
$N_2O/(N_2O+N_2)$	Linear	**0.546**	**<0.001**	**-138.1**	**1.07**	✔
	Quadratic	0.561	<0.001	-139.2	0.00	

Supplementary Table 6. Summary for the linear and quadratic models predicting the relationship between soil pH and the coarse abundances of soil *nirK*, *nirS*, and *nosZI*, and the (*nirK*+*nirS*)/*nosZI* ratio. Linear regression model with two-sided test was used for the statistical analysis, and adjusted R-squared was used. Models are ranked by significance, AICc and parsimony. AICc measures the relative goodness of fit of a given model; the lower its value, the more likely the model is correct. ΔAICc denotes the difference between the AICc of each model and that of the best model.

Denitrifiers	Model	Adjusted R^2	*P* value	AICc	Delta AICc	Selected Model
Soil pH vs. *nirK*	Linear	0.011	<0.001	4590.6	39.89	
	Quadratic	**0.041**	**<0.001**	**4550.7**	**0.00**	✔
Soil pH vs. *nirS*	Linear	0.001	0.162	4643.7	19.14	
	Quadratic	**0.017**	**<0.001**	**4624.5**	**0.00**	✔
Soil pH vs. *nosZI*	Linear	0.000	0.844	4276.7	2.77	**undetermined**
	Quadratic	0.002	0.090	4273.9	0.00	
Soil pH vs. *(nirK+nirS)/nosZI*	Linear	0.012	<0.001	3255.2	40.34	
	Quadratic	**0.047**	**<0.001**	**3214.9**	**0.00**	✔

Supplementary Table 7. Summary for the linear and quadratic models predicting the relationship between soil pH and the averaged abundances of soil *nirK*, *nirS*, and *nosZI*, and the ratio of (*nirK*+*nirS*)/*nosZI*. Linear regression model with two-sided test was used for the statistical analysis, and adjusted R-squared was used. Models are ranked by significance, AICc and parsimony. AICc measures the relative goodness of fit of a given model; the lower its value, the more likely the model is correct. ΔAICc denotes the difference between the AICc of each model and that of the best model.

Denitrifiers	Model	Adjusted R^2	*P* value	AICc	Delta AICc	Selected Model
Soil pH vs.	Linear	0.004	0.276	160.1	31.82	
nirK	Quadratic	**0.448**	**<0.001**	**128.2**	**0.00**	✔
Soil pH vs.	Linear	0.270	**<0.001**	156.3	37.26	
nirS	Quadratic	**0.633**	**<0.001**	**119.0**	**0.00**	✔
Soil pH vs.	Linear	0.078	0.021	134.3	3.24	**undetermined**
nosZI	Quadratic	0.149	0.005	131.1	0.00	
Soil pH vs.	Linear	0.016	0.173	136.0	26.21	
(*nirK*+*nirS*)/*nosZI*	Quadratic	**0.403**	**<0.001**	**109.8**	**0.00**	✔

Supplementary References:

1. Wang, Y. Guo, J. Vogt, R. D., Mulder, J., Wang, J. & Zhang, X. Soil pH as the chief modifier for regional nitrous oxide emissions: new evidence and implications for global estimates and mitigation. *Glob. Change Biol.* **24,** e617–e626 (2018).
2. Cui, X. et al. Global mapping of crop-specific emission factors highlights hotspots of nitrous oxide mitigation. *Nat. Food* **2,** 886–893 (2021).
3. Jones, C. M., Putz, M., Tiemann M. & Hallin S. Reactive nitrogen restructures and weakens microbial controls of soil N_2O emissions. *Commun. Biol.* **5,** 273 (2022).
4. Guo, J. H. et al. Significant acidification in major Chinese croplands. *Science* **327**, 1008−1010 (2010).
5. Yu, Z., Liu, J. & Kattel, G. Historical nitrogen fertilizer use in China from 1952 to 2018. *Earth Syst. Sci. Data* **14,** 5179–5194 (2022).
6. Ouyang, Y., Evans, S. E., Friesen, M. L. & Tiemann, L. K. Effect of nitrogen fertilization on the abundance of nitrogen cycling genes in agricultural soils: A meta-analysis of field studies. *Soil Biol. Biochem.* **127,** 71–78 (2018).
7. Stark, J. M. & Hart, S. C. High rates of nitrification and nitrate turnover in undisturbed coniferous forests. *Nature* **385,** 61–64 (1997).
8. O'Mara, F. P. The role of grasslands in food security and climate change. *Ann. Bot.* **110,** 1263–1270 (2012).
9. Buisson, E., Archibald, S., Fidelis, A. & Suding, K. N. Ancient grasslands guide ambitious goals in grassland restoration. *Science* **377**, 594–598 (2022).
10. Dangal, S. R. S. et al. Global nitrous oxide emissions from pasturelands and rangelands: magnitude, spatiotemporal patterns and attribution. *Glob. Biogeochem. Cycles* **33,** 200–222 (2019).

11. Chang, J. et al. Climate warming from managed grasslands cancels the cooling effect of carbon sinks in sparsely grazed and natural grasslands. *Nat. Commun.* **12,** 118 (2021).

12. Wei, Y., Jing, X., Su, F., Li, Z., Wang, F. & Guo, H. Does pH matter for ecosystem multifunctionality? An empirical test in a semi-arid grassland on the Loess Plateau. *Funct. Ecol.* **36,** 1739–1753 (2022).
