# Supplementary material for: Intermediate soil acidification induces highest nitrous oxide emissions
Source: Nat Commun. 2024 Mar 27;15:2695. doi: 10.1038/s41467-024-46931-3 (PMC10973416; doi:10.1038/s41467-024-46931-3)
Supplement: Supplementary file 3 — Description of Additional Supplementary Files [file 41467_2024_46931_MOESM3_ESM.pdf]

Description of Additional Supplementary Files

Title: Supplementary Data 1

Description: Meta-analysis 1 (Global synthesis of effects of N input and soil pH on N_2O emission factors).

Title: Supplementary Data 2

Description: Three field experiments with acid additions to determine soil acidification effects on nitrifying and denitrifying microorganisms, and soil N_2O emissions.

Title: Supplementary Data 3

Description: Meta-analysis 2 (Global relationship between soil pH and denitrifying microorganisms).
